# Study on the Susceptibility of Some Almond (*Prunus dulcis*) Cultivars to the Pathogen *Diaporthe amygdali*

**DOI:** 10.3390/plants15010165

**Published:** 2026-01-05

**Authors:** Pompea Gabriella Lucchese, Naïma Dlalah, Amélie Buisine, Franco Nigro, Stefania Pollastro, Henri Duval

**Affiliations:** 1 Department of Soil, Plant and Food Sciences, University of BariAldo Moro, 70126 Bari, Italy; franco.nigro@uniba.it (F.N.); stefania.pollastro@uniba.it (S.P.); 2 Centro di Ricerca, Sperimentazione e Formazione in Agricoltura Basile Caramia (CRSFA), 70010 Locorotondo, Italy; 3 Institut National de Recherche pour l’Agriculture, l’Alimentation et l’Environnement (INRAE), Unité de Génétique et Amélioration des Fruits et Légumes (GAFL), F-84143 Montfavet, France; naima.dlalah@inrae.fr (N.D.); amelie.buisine@inrae.fr (A.B.)

**Keywords:** canker disease, field inoculation methods, phylogenetic analysis, *Phomopsis amygdali*

## Abstract

*Diaporthe amygdali* Delacr. is a phytopathogenic fungus of considerable agronomic importance, responsible for branch canker in almond (*Prunus dulcis* [Mill.] D.A. Webb) and peach (*Prunus persica* L.) trees. It represents a major phytosanitary threat to almond cultivation in Europe, particularly in Mediterranean regions. Almond is currently among the most rapidly expanding perennial crops, with cultivated areas increasing as a result of the introduction of new cultivars and the adoption of improved agronomic practices. The objectives of this study were to isolate and identify fungal pathogens from infected almond samples collected in France through multilocus phylogenetic analyses (ITS, *tef1-α*, *his3*, *tub2*, *cal* genes) combined with morphological characterization; evaluate the susceptibility of 18 almond genotypes, using ‘Ferragnès’ and ‘Texas’ as reference standards for susceptibility and tolerance, respectively; and compare three field inoculation methods. All isolates were identified as *D. amygdali*. The varietal screening identified marked differences in resistance among the tested cultivars. In particular, ‘Ferrastar’, ‘R1877’, ‘R1413’, and ‘R1542’ exhibited high levels of resistance, whereas ‘Tuono’, ‘Guara’, and ‘R1568’ showed susceptibility comparable to that of ‘Ferragnès’, which was used as the susceptible control. Among the inoculation methods evaluated, the mycelial plug technique proved to be the most consistent and reliable, outperforming both conidial suspension inoculation and the toothpick method coated with mycelium. These findings further confirm the genetic resistance of the cultivars ‘Ferrastar’ and ‘Ardèchoise’ to branch canker across different growing conditions, supporting their suitability for use in breeding and genetic improvement programs.

## 1. Introduction

Almond (*Prunus dulcis* [Mill.] D.A. Webb) production has experienced remarkable global growth in recent years, driven by both agronomic and economic factors. Over the past 15 years, the global context has remained favorable for almond cultivation [1]. The rising demand is largely attributed to the well-documented health benefits associated with almond consumption. Almonds are rich in vitamin E, protein, monounsaturated and polyunsaturated fatty acids, magnesium, potassium, and dietary fiber—nutrients linked to a reduced risk of cardiometabolic disorders [2]. In addition, expanding markets in Asia and other regions have further stimulated demand and elevated market prices [3].

As a consequence of these global trends, almond production systems are undergoing a fundamental transformation in the Mediterranean region. Once considered a marginal, rain-fed crop, almond is now cultivated under intensified and profitable systems, incorporating new varieties and improved management practices aimed at enhancing yield and profitability [4].

In France, although almond cultivation remains limited in extent, farmers’ interest is increasing, particularly in southern regions traditionally devoted to cereals, lavender, vegetables, and viticulture. As of 2023, France’s almond acreage has reached 2330 hectares [5]—an infinitesimal share compared to the total almond-growing area in Europe, which spans approximately 915,561 hectares [5]. Nonetheless, the growing investment in almond orchards in southern France signals an important shift in agricultural priorities and potential for future expansion.

Globally, almond diseases have been extensively investigated [6,7,8,9]. Major diseases include red leaf blotch (*Polystigma amygdalinum* P.F. Cannon), shot hole (*Wilsonomyces carpophilus [Lév.] Adask*., J.M. Ogawa & E.E. Butler), brown rot and blossom blight (*Monilinia* spp.), and leaf curl (*Taphrina deformans [Berk.] Tul*.) [10,11]. Furthermore, relatively new diseases affecting almond—such as anthracnose, mostly caused by *Colletotrichum godetiae* Neerg [12], trunk and branch canker pathogens [9,13,14], as well as Almond Leaf Scorch Disease caused by different subspecies of *Xylella fastidiosa* Wells et al. (1987) [15,16,17]—have also been reported.

Among these, particular attention in recent decades has focused on fungal canker pathogens, owing to their capacity to substantially reduce orchard longevity and productivity [13,14,18,19]. The disease caused by *Diaporthe amygdali* (Delacroix) Udayanga, Crous & K.D. Hyde is characterized by the rapid desiccation of shoots, flowers, and leaves following infections that typically occur between late winter and early spring [20,21,22,23]. Infected buds develop sunken, reddish-brown necrotic lesions that evolve into cankers exuding small quantities of gum [24]. As lesions expand, they girdle twigs, causing distal shoot dieback and general canopy wilting. The fungus overwinters in the bark of the cankers [20].

Although *D. amygdali* primarily infects members of the genus *Prunus*—including almond, peach, and nectarine—its pathogenicity has been widely documented worldwide [3,25,26,27,28,29,30,31,32,33,34]. The pathogen has also been reported from several unrelated hosts, such as grapevine in South Africa [35], Japanese andromeda in the United States [36], Asian pear and walnut in China [37], and blueberry in Portugal [38], demonstrating its polyphagous nature.

Lalancette and co-authors [39,40] elucidated key aspects of disease development on peach plantations in New Jersey (USA), showing that *D. amygdali* primarily penetrates through wounds formed during leaf abscission in autumn or shoot emergence and flower/fruit drop in spring. Infections can also arise directly through young shoots [41]. The incubation period from infection to visible canker development is approximately one month. Meteorological conditions strongly influence disease expression: in mild autumns and winters, cankers develop rapidly, leading to shoot dieback in early spring; conversely, cold winters delay symptom expression, resulting in shoot death during the following summer. Under favorable conditions, *D. amygdali* produces pycnidia within the cankers, releasing α-conidia—the predominant form found on almond—and occasionally β-conidia [22,41]. The formation and release of conidia, often extruded as cirri, are regulated by environmental parameters, particularly temperature and relative humidity [40,42]. Fungal development is slow at 5 °C but accelerates above 10 °C [24]; growth occurs across a broad temperature range (0–37 °C), with optimal growth around 19–20 °C. Maximum conidial production occurs between 22 °C and 23 °C under a relative humidity above 95% sustained for at least 16 h [40]. These environmental conditions are critical to disease epidemiology, as they not only promote conidial growth and dispersal but also delay the natural healing of wounds caused by leaf abscission. Rain events further facilitate pathogen dissemination, as conidia are dispersed by water splashing to adjacent tissues and trees, where they germinate on moist surfaces at temperatures between 5 °C and 36 °C [24,42].

Over the past decade, research on the vulnerability of almond cultivars to fungal diseases has increased substantially compared with earlier years. In one of the first systematic studies conducted in Spain, Egea et al. (1984) [43] evaluated the susceptibility of 81 almond varieties to red leaf blotch. In California (USA), Gradziel and Wang [44] investigated the susceptibility of several cultivars to *Aspergillus flavus* Link, while Diéguez-Uribeondo et al. (2011) [45] assessed the response of four cultivars to *Colletotrichum acutatum* Simmonds. In Australia, field evaluations of 34 almond cultivars determined their susceptibility to *Tranzschelia discolor* (Fuckel) Tranzschel & M.A. Litv, the causal agent of rust, under both natural and artificial inoculation conditions [46]. More recently, López-Moral et al. (2019) [12] examined 19 Spanish cultivars for their sensitivity to *C. acutatum* and *C. godetiae*, and subsequent studies have evaluated the response of early- and late-flowering cultivars to leaf pathogens such as *Monilinia laxa* (Aderhold & Ruhland) Honey, *P. amygdalinum*, *T. deformans*, and *W. carpophilus* [10,11]. Specific studies on the susceptibility of almond cultivars to *D. amygdali* have also been conducted in several countries. In Chile, Besoain et al. (2000) [27] found that cultivars ‘Nonpareil’ and ‘Price’ were more susceptible than ‘Carmel’ under artificial inoculation. In Portugal, the local cultivar ‘Barrinho Grado’ showed greater tolerance than ‘Ferragnès’ [28]. In Spain, Vargas and Miarnau [47] evaluated more than 70 cultivars and 36 breeding selections under natural field conditions, revealing a high overall susceptibility to *Diaporthe* dieback. In Hungary, Varjas et al. (2017) [23] assessed 162 almond genotypes over four consecutive years, identifying 31 cultivars with very high levels of tolerance. In particular, ‘Budatétényi-70’ and ‘Tétényi keményhéjú’ were significantly more tolerant than the other Hungarian genotypes, suggesting considerable genetic variability in disease resistance.

The objectives of the present study were to (i) characterize the identity and diversity of *Diaporthe* species associated with canker disease in almond orchards in southern France; (ii) compare three field inoculation methods—conidial suspension, mycelial plug, and toothpicks colonized with mycelium; and (iii) assess the susceptibility of 18 almond genotypes of French and Italian origin, using ‘Ferragnès’ and ‘Texas’ as susceptible and tolerant controls, respectively. This study differs from previous investigations in that it aimed to evaluate the performance of different almond genotypes against multiple pathogens isolates under field conditions closely resembling natural infection. The experimental design incorporated both commercial cultivars already reported in the literature and hybrid selections developed at the INRAE Research Centre.

## 2. Results

### 2.1. Isolation and Identification of the Pathogen 

A total of six isolates were obtained from one-year-old branches with obvious symptoms of cankers on the nodes (Table 1). The two strains, M2 and M17, were collected in 2018 within the framework of the French Plant Protection Product (PPP) resistance monitoring plan, as part of the SBT (Biological Monitoring of the Territory) programme, and were isolated by the USC CASPER research unit of ANSES (Lyon, France).

**Figure 1:**
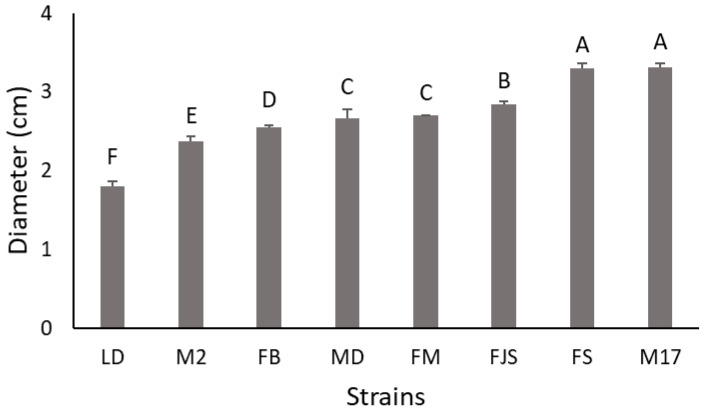
Colony diameter of *Diaporthe amygdali* isolates after 4 days of incubation at 25 ± 1 °C. Statistically significant differences among isolates are indicated by different letters according to Tukey’s test (*p* < 0.001). Data represent mean ± standard error (n = 3).

**Figure 2:**
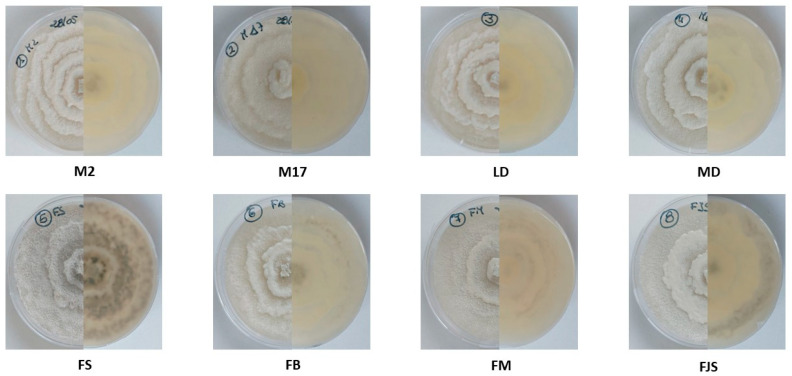
Colony morphology of *Diaporthe amygdali* isolates cultured on PDA after 10 days of incubation at 25 ± 1 °C. For each isolate, the left half of the plate shows the top view, and the right half shows the bottom view of the same colony. The panel includes two reference strains (M2 and M17) and six field isolates (LD, MD, FS, FB, FM, and FJS). The images represent typical growth patterns across isolates.

All isolates exhibited similar morphological characteristics when cultured on PDA, with the exception of isolate LD, which showed a slower growth rate. After incubation at approximately 25 ± 1 °C for 4 days, isolate LD reached a mean colony diameter of 1.8 ± 0.1 cm (mean ± standard error), whereas the other isolates attained an average diameter of 2.8 ± 0.2 cm (mean ± standard error) (Figure 1). As illustrated in Figure 2 the differences in colony diameter among the isolates were statistically significant. Notably, isolates FS and M17 displayed the highest growth rates, with mean colony diameters of 3.3 ± 0.1 cm (mean ± standard error), for both.

**Figure 3:**
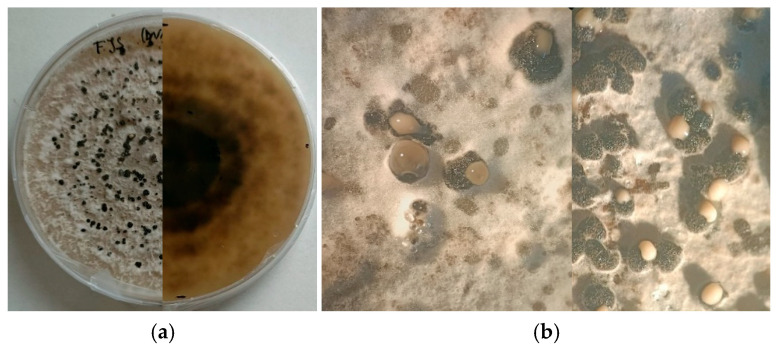
(**a**) Fifteen-day-old culture of *Diaporthe amygdali* grown on PDA at 25 ± 1 °C under a 12 h UV-light photoperiod; (**b**) Conidial masses exuding from pycnidia developed on PDA after 20 days of incubation under the same conditions.

Furthermore, differences in radial growth among the isolates were assessed by calculating the daily growth rate on agar media. As shown in Figure S1, isolate LD exhibited the lowest growth rate (0.8 cm day^−1^), whereas isolate FS showed the highest growth rate (1.1 cm day^−1^). However, despite this apparent variation, the differences were not statistically significant (see Figure 2 and Figure 3).

**Figure 4:**
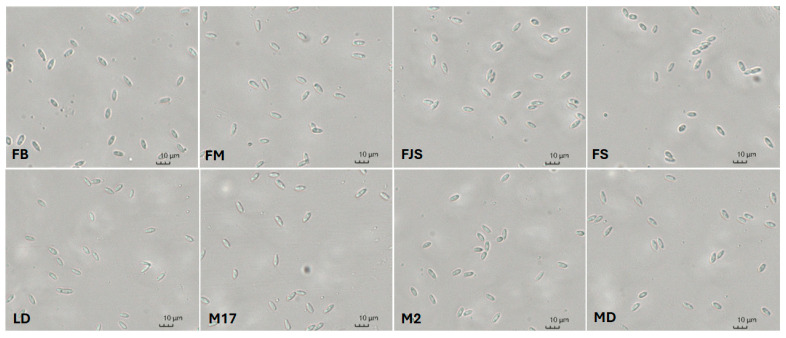
Microscopic view of α-conidia produced by different *Diaporthe amygdali* isolates (FB, FM, FS, FJS, LD, MD, M2, and M17) observed under 400× magnification.

Approximately 10 conidia were measured per replicate, resulting in a total of 30 conidia per isolate. Overall, conidial dimensions ranged from 6.6 to 9.1 × 2.3 to 3.1 µm. Statistically significant differences among isolates were detected, with isolates FJS and MD exhibiting significantly greater conidial length and width compared with the other isolates (8.7 ± 0.4 × 3.1 ± 0.1 µm and 9.1 ± 0.4 × 2.9 ± 0.1 µm, respectively; Table 2). All isolates produced α-conidia with the same morphology—fusiform, hyaline, and biguttulate (Figure 4). No β-conidia were detected. Approximately 10 conidia were measured per replicate, for a total of 30 conidia per isolate.

Single-locus phylogenetic analyses were performed separately for *cal*, *his3*, ITS, *tub2*, and *tef1-α* (Figure S2A–E) using the Maximum Likelihood method. The individual loci exhibited heterogeneous phylogenetic signal. The ITS locus recovered a main clade with high bootstrap support but showed limited resolution among closely related taxa. The *cal* and *his3* loci provided moderate resolution and partially supported the clustering of *Diaporthe amygdali* isolates. In contrast, the *tub2* phylogeny displayed topological incongruence, with *D. amygdali* isolates failing to form a monophyletic group and showing inconsistent placement relative to other loci. The *tef1-α* locus provided comparatively improved clustering of the examined isolates, although some internal relationships remained weakly supported. Minor differences in taxon sampling among single-locus trees were due to the absence of some loci for specific reference strains (Table S3).

**Figure 5:**
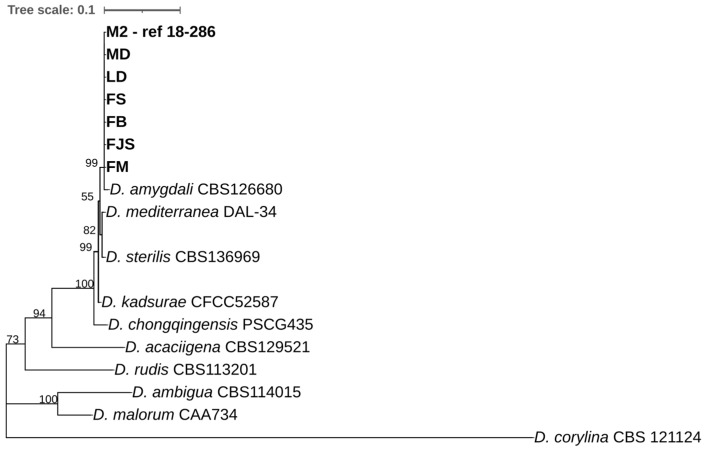
Phylogenetic tree showing the relationships among *Diaporthe* species and *D. amygdali* isolates collected from different regions of southern France, based on concatenated sequences of five loci (*ITS*, *tef1-α*, *cal*, *his3*, and *tub2*). The tree was constructed using the Maximum Likelihood (ML) method under the Tamura–Nei substitution model, with a scale bar representing 0.1 nucleotide substitutions per site. The numbers at the nodes represent the bootstrap support expressed as a percentage.

A multi-locus phylogenetic analysis based on concatenated sequences of ITS, *tef1-α*, *cal*, *his3*, and *tub2* was subsequently conducted using the Maximum Likelihood method under the Tamura–Nei substitution model (Figure 5). In the resulting tree, all isolates obtained in this study (FB, FM, MD, LD, FS, FJS, and M2) clustered together with the reference strain *Diaporthe amygdali* CBS 126680, forming a single clade supported by moderate bootstrap values, and clearly separated from other *Diaporthe* species. The tree was rooted with *Diaporthella corylina* CBS 121124 as the outgroup. The analysis based on the Maximum Likelihood method revealed that all strains examined in this study (FB, FM, MD, LD, FS, FJS, and M2) clustered within a single clade together with *Diaporthe amygdali* CBS 126680, supported by a 99% bootstrap value. This clade was phylogenetically related to *D. mediterranea* DAL-34 and *D. sterilis* CBS 136969, with bootstrap support ranging from 55% to 99%. Other reference species, including *D. kadsurae*, *D. chongqingensis*, *D. acacigena*, *D. rudis*, *D. ambigua*, and *D. malorum*, formed distinct and well-supported clades (bootstrap 73–100%). The tree was rooted with *Diaporthella corylina* CBS 121124 as the outgroup (Figure 5).

### 2.2. Inoculation Methods

**Figure 6:**
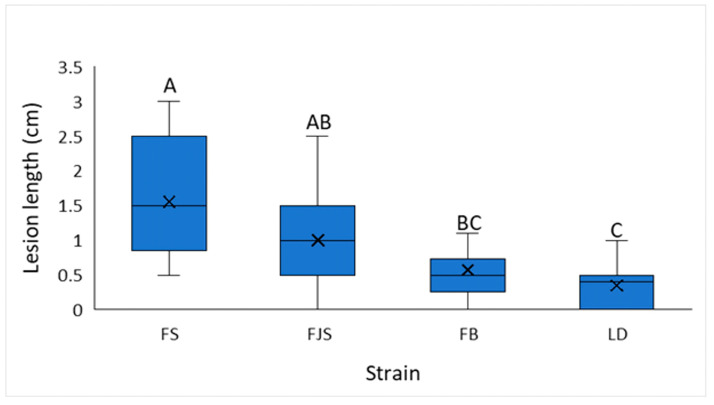
Lesion length (cm) induced by different *Diaporthe amygdali* isolates using the conidial suspension method, 90 days after inoculation, considering all varieties reported in Table 2. Different letters indicate statistically significant differences according to Tukey’s test (*p* < 0.01) (n = 54).

Four *D. amygdali* isolates (FB, FJS, FS, and LD) were inoculated using a conidial suspension. The results presented in Figure 6 show the lesion lengths produced by the different isolates. The LD isolate exhibited consistently slower growth on PDA, which was also reflected in reduced symptom development on inoculated tissues, with a mean lesion length of 0.4 ± 0.1 cm (mean ± standard error). Conversely, isolate FS produced statistically significantly longer lesions than all other isolates, with a mean length of 1.5 ± 0.2 cm (mean ± standard error). Isolates FJS and FB showed intermediate virulence, causing lesions of 1.0 ± 0.2 cm and 0.6 ± 0.1 cm, respectively (mean ± standard error).

**Figure 7:**
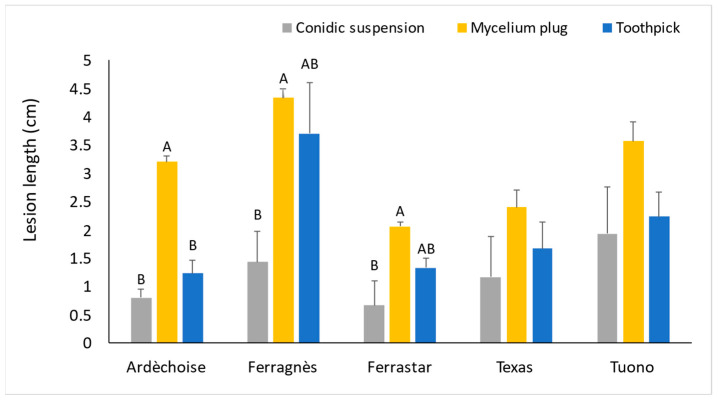
Lesion length (cm) induced by different inoculation methods using isolate FS, evaluated 90 days after inoculation. Data are reported as mean ± standard error (n = 3). Different letters indicate statistically significant differences among inoculation methods within each cultivar, according to Tukey’s test (*p* < 0.05). Ardéchoise, Ferragnès, Ferrastar, Texas and Tuono are almond (*Prunus dulcis*) cultivars.

Regarding the inoculation methods, and considering only the cultivars ‘Ardèchoise’, ‘Ferragnès’, ‘Ferrastar’, ‘Texas’, and ‘Tuono’ inoculated with isolate FS (Figure 7), the results indicate that the mycelial plug method was the most effective. This technique produced significantly longer lesions, with mean lesion lengths of 3.2 ± 0.11 cm, 4.3 ± 0.17 cm, and 2.0 ± 0.07 cm in ‘Ardèchoise’, ‘Ferragnès’, and ‘Ferrastar’, respectively (mean ± standard error). These values were significantly higher than those obtained with the toothpick inoculation method (1.2 ± 0.2 cm, 3.7 ± 0.9 cm, and 1.3 ± 0.2 cm for the same cultivars) and with the conidial suspension technique (0.8 ± 0.2 cm, 1.4 ± 0.5 cm, and 0.7 ± 0.4 cm, respectively).

**Figure 8:**
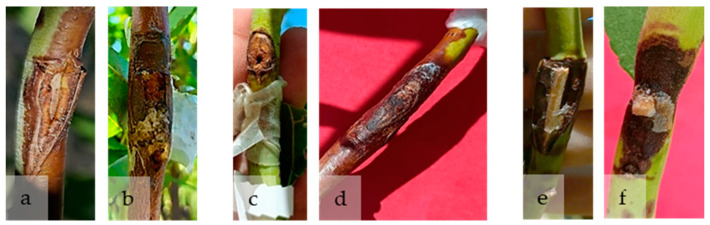
Lesions caused by *Diaporthe amygdali* using three different inoculation methods. (**a**) Negative control inoculated with sterile distilled water; (**b**) lesions observed in September following inoculation with a conidial suspension; (**c**) negative control inoculated with a sterile PDA plug; (**d**) lesions observed in September following inoculation with a mycelial plug; (**e**) negative control inoculated with a sterile toothpick; (**f**) lesions observed in September, respectively, following inoculation with a toothpick colonized by mycelium.

Moreover, the experiment was easier to perform using a toothpick or a plug colonized by mycelium than with the conidial suspension, as the margin of error introduced by cutting could influence the final lesion size (Figure 8).

### 2.3. Varietal Susceptibility

**Figure 9:**
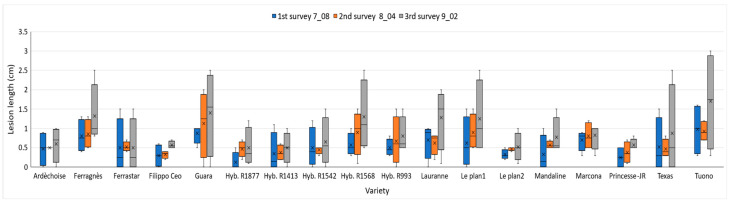
Mean lesion length (cm) recorded in three consecutive evaluations: 7 July (blue), 4 August (orange), and 2 September (gray). (n = 12). Names reported on the x-axis refer to almond cultivars.

Figure 9 presents the results of the three assessments conducted at 30, 60, and 90 days after inoculation. Notably, hybrid R1877 exhibited a low mean lesion length of 0.5 ± 0.1 cm (mean ± standard error), comparable to those observed in ‘Ferrastar’, hybrid R1413, and hybrid R1542, which all showed lesion lengths ≤ 0.7 cm.

In contrast, ‘Tuono’, ‘Guara’, ‘Ferragnès’, and ‘R1568’ were highly susceptible, showing mean lesion lengths of 1.7 ± 0.3 cm, 1.4 ± 0.3 cm, 1.3 ± 0.2 cm, and 1.3 ± 0.2 cm, respectively (mean ± SE). Intermediate responses were observed in the cultivars ‘Lauranne’ and ‘Le Plan 1’, which exhibited mean lesion lengths of 1.3 ± 0.2 cm (mean ± standard error), for both. Individual analyses of variance (ANOVA) for each evaluation did not reveal any statistically significant differences among the varieties (*p* > 0.05) (Figure 9).

**Figure 10:**
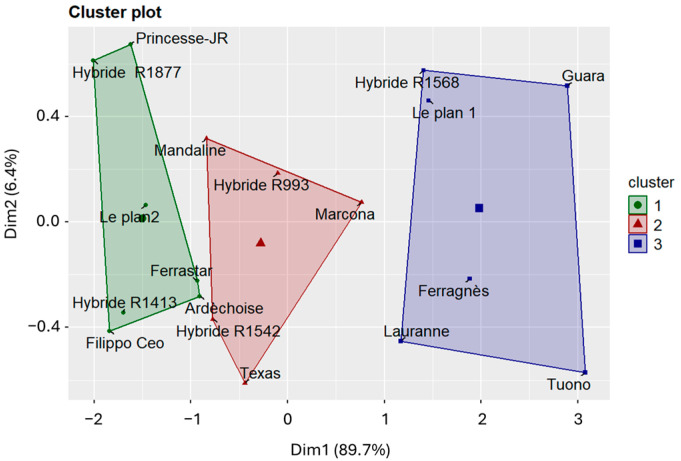
k-means cluster plot of 18 almond varieties based on lesion length measurements obtained from three disease evaluation surveys. The three clusters represent groups of varieties with different levels of susceptibility: Cluster 1 (green)—tolerant; Cluster 2 (red)—intermediate; and Cluster 3 (blue)—susceptible. Axes (Dim1 and Dim2) correspond to the first two principal components, which together explain approximately 96% of the total variance. All labels represent cultivar or hybrid names and are reported as proper names.

However, clustering analyses (k-means, NbClust) and principal component analysis (PCA)-based dimensionality reduction revealed a clear pattern in the data. Because the principal components are orthogonal, they provide a feature space that is particularly well suited for k-means clustering, enabling more stable and reproducible cluster assignment. The optimal number of clusters was determined using the NbClust package. (Figure 10). The results highlighted the presence of three well-defined clusters of varieties, each exhibiting a consistent pattern of symptom expression. The mean lesion lengths for each cluster are presented in Table S1. Cluster 1 comprised varieties showing intermediate and stable behavior over time; Cluster 2 included tolerant varieties, characterized by low lesion values and limited progression; and Cluster 3 contained the most susceptible varieties, displaying larger lesions and a pronounced increase over time.

**Figure 11:**
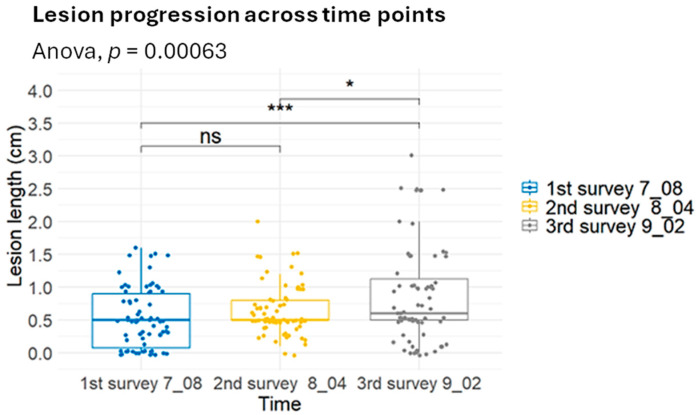
Progression of lesion length (cm) across three evaluation periods (8 July, 4 August, and 2 September). Box plots display the median, interquartile range, and individual data points. One-way ANOVA (*p* = 0.00063) indicated significant differences among time points, with *** denoting *p* < 0.001, * denoting *p* < 0.05, and ns indicating non-significant comparisons.

The mean lesion length showed a consistent increase over the three survey periods (Figure 11). Lesion lengths were comparatively short and not significantly different (*p* > 0.05) during the first and second evaluations (8 July and 4 August). However, a marked and statistically significant increase was observed in the third survey (2 September), as confirmed by one-way ANOVA (*p* = 0.00063) and post hoc tests (*p* < 0.001). These results indicate a clear temporal progression of the disease, with lesion development intensifying significantly during the latter part of the observation period.

## 3. Discussion

Morphological and molecular characterization of isolates obtained from naturally infected almond samples collected across multiple orchards in southern France confirmed that all isolates were attributable exclusively to *Diaporthe amygdali*. Morphological observations revealed that one isolate (LD) exhibited noticeably slower growth than the other isolates. In contrast, isolates FS, FJS and M17 showed markedly higher growth rates and abundant pycnidia formation.

Regarding conidial morphology, only α-type conidia were observed, with no detectable variation among isolates, consistent with previous reports [48,49,50]. These morphological findings, together with multilocus sequence data (ITS, *tef1-α*, *cal*, *his3*, and *tub2*), confirmed the identity of all isolates as *D. amygdali*, in full agreement with the results of Gusella et al. (2023) [48] in Sicily (South Italy), who also found that almond-derived isolates clustered closely with reference strains of this species. Multilocus phylogenetic analyses are widely recognized as the standard approach for molecular characterization and species delimitation within the genus *Diaporthe*. Numerous studies have relied primarily on concatenated multilocus datasets to infer species-level phylogenetic relationships, reflecting the limited resolving power of individual loci when analyzed independently [9,31,48,51,52,53,54]. The integration of multiple loci enhances phylogenetic resolution and robustness, providing a more reliable framework for accurate species delimitation than single-locus analyses.

With respect to inoculation techniques, the mycelial plug method proved to be the most effective, as it produced rapid symptom development and facilitated detection. This observation aligns with previous studies reporting similar outcomes [3,21,31,48]. The toothpick inoculation method, in which the toothpick is colonized by fungal mycelium, was moderately effective, whereas the conidial suspension technique, although simpler to perform, was the least efficient. In the latter case, lesion size could be affected by the dimensions of the incision, and symptom expression was delayed (approximately 60 days post-inoculation).

Comparative analyses of *D. amygdali* isolates revealed significant differences in pathogenicity. The LD isolate produced statistically smaller lesions than the FS isolate (Figure 6), consistent with its slower growth and lower pycnidia and conidia production in vitro. Conversely, isolate FS was characterized as the most aggressive, producing the largest lesions and exhibiting the fastest mycelial growth on PDA, followed closely by M17.

Regarding cultivar susceptibility, all tested varieties developed cankers at the inoculation point, although lesion length varied. While these differences were not statistically significant, symptom onset occurred earlier in some cultivars, whereas others maintained smaller, more stable cankers, indicating greater tolerance to the pathogen. This pattern is illustrated in Figure 10: varieties belonging to Cluster 1 (green)—‘Ardèchoise’, ‘Ferrastar’, ‘Hybride R1877’, ‘Filippo Ceo’, ‘Hybride R1413’, ‘Le Plan 2’, and ‘Princesse-JR’—displayed smaller, stable lesions throughout the experiment. The result for ‘Filippo Ceo’ is consistent with the findings of Piglionica et al. (1967) [49] and Catalano et al. (2025) [50], who also reported this cultivar among those developing the shortest cankers.

Cluster 2 included ‘Hybride R1542’, ‘Hybride R993’, ‘Mandaline’, ‘Marcona’, and ‘Texas’, which showed intermediate lesion lengths that remained stable over time. The performance of ‘Texas’ corroborates the results of Catalano et al. (2025) [50], while ‘Marcona’ showed an intermediate susceptibility pattern similar to that described by Beluzán et al. (2022) [3]. Cluster 3 comprised ‘Ferragnès’, ‘Guara’, ‘Hybride R1568’, ‘Lauranne’, ‘Le Plan 1’, and ‘Tuono’, which developed longer and more rapidly expanding cankers, indicating higher susceptibility. These results are consistent with the field-based sensitivity assessment conducted by Vargas and Miarnau [46], who classified the cultivars ‘Ardèchoise’ and ‘Ferrastar’ as tolerant, and ‘Ferragnès’, ‘Lauranne’, ‘Tuono’, and ‘Guara’ as susceptible. However, in contrast to our findings, they reported the cultivar ‘Texas’ to exhibit a higher level of tolerance.

Overall, this study provides valuable insights into the responses of different almond cultivars to *D. amygdali*, a pathogen that has re-emerged in recent years and poses a growing threat to almond production in the Mediterranean region. The identification of potentially tolerant cultivars under Mediterranean conditions—corroborating findings from southern Italy and Spain—highlights the importance of these genotypes as potential sources of resistance in future breeding and selection programs. Although inoculation with conidial suspensions proved less effective in symptom onset, this method most closely mimics natural infection processes in the field. Therefore, it remains a valuable approach for assessing cultivar responses under conditions approximating natural pathogen–host interactions.

## 4. Material and Methods

### 4.1. Isolation, Morphological and Molecular Characterization

Branches showing symptoms indicative of *Diaporthe amygdali* infection—such as shoot blight, cankers, and gum exudation—were collected from almond orchards located in the Provence region of southern France during April and May. Samples were taken from various positions within the canopy, focusing on areas where symptoms were most evident. Each specimen was placed in a humid chamber and incubated at ambient temperature for approximately two weeks to promote the development of cirri. Once cirrus formation was observed, exudates were aseptically collected using a sterile needle loop and transferred onto Petri dishes containing potato dextrose agar (PDA; Difco Laboratories, Detroit, MI, USA) supplemented with 0.5 g/L streptomycin sulfate (Sigma-Aldrich, St. Louis, MO, USA) to inhibit bacterial growth. Plates were incubated at 25 ± 1 °C [38] for up to two weeks and monitored regularly for fungal development and possible contamination.

Fungal colonies exhibiting morphological characteristics consistent with *Diaporthe* spp. were subcultured onto fresh PDA plates to obtain pure cultures. Single-spore isolation was performed by transferring individual conidia onto new PDA plates under a stereomicroscope in sterile conditions. The purified isolates were incubated at 25 ± 1 °C in the dark for 7–10 days as described by Hilario et al. (2021) [38] and subsequently stored at 4 °C for further morphological and molecular analyses.

Following purification, the isolates were subjected to detailed morphological characterization. To stimulate sporulation and allow examination and measurement of reproductive structures, each isolate was cultured on PDA and incubated at 25 ± 1 °C under a 12 h light/dark photoperiod with UV illumination for 15 days. Conidial measurements were conducted by mounting spores in sterile water on microscope slides after growth on PDA. Observations and measurements were performed using an Olympus BH2 BHS-312 trinocular microscope (Olympus Corporation, Tokyo, Japan) at 400× magnification. Colony growth was assessed by measuring the radial expansion of the mycelium on PDA after 4 and 10 days of incubation. For accuracy and reproducibility, all measurements were performed in triplicate for each isolate.

A total of eight isolates were used for molecular characterization, including six isolates obtained in this study and two reference isolates from Corsica. Genomic DNA was extracted according to the protocol described by [55]. PCR amplification of five conserved genomic regions was carried out using primer sets (Table S2). The internal transcribed spacer (ITS) region of the nuclear ribosomal RNA operon was amplified using primers ITS1 and ITS4 [56]. A portion of the translation elongation factor 1-α (*tef1-α*) gene was amplified using primers EF1-688F and EF1-1251R [57]; *tef1-α* encodes an elongation factor involved in regulating the rate and fidelity of protein synthesis. Partial sequences of the β-tubulin (*tub2*) gene were amplified using primers BtCadF and BtCadR [58]; *tub2* encodes β-tubulin, a key structural component of the cytoskeleton. The histone H3 (*his3*) gene was amplified with primers CYLH3F and H3-1b [59]; *his3* encodes histone H3, which plays a central role in chromatin organization and epigenetic regulation of gene expression. The calmodulin (*cal*) gene was amplified using primers CL1C and CL2C [60]; *cal* encodes a Ca^2+^-dependent regulatory protein involved in intracellular signal transduction.

Each 50 µL PCR reaction contained 10 µL of 5× Green GoTaq^®^ Flexi Buffer (Promega Corporation, Madison, WI, USA), 3 µL of 25 mM MgCl_2_, 0.4 µL of GoTaq^®^ DNA Polymerase (Promega Corporation, Madison, WI, USA), 1 µL of each primer at 20 µM (except for the *his3* primers, for which 0.5 µL of each was used), and 4 µL of genomic DNA at dilutions ranging from 1:800 to 1:20. The final reaction volume was adjusted with nuclease-free water.

PCR amplifications were performed in an Eppendorf 5341 Mastercycler epGradient (Eppendorf AG, Hamburg, Germany) thermal cycler under the following conditions: initial denaturation at 95 °C for 5 min; followed by 35 cycles of denaturation at 95 °C for 30 s, annealing for 30 s at primer-specific temperatures (55 °C for ITS1-4 and *tef1-α*, 58 °C for *cal*, 50 °C for BtCadF–BtCadR, and 59 °C for *his3*), and extension at 72 °C for 40 s; with a final extension at 72 °C for 10 min.

Amplified products (10 µL) were separated by electrophoresis on 1.5% (*w*/*v*) agarose gels and visualized under UV illumination.

Raw sequence data for each isolate were analyzed using CLC Main Workbench v8 (QIAGEN, Hilden, Germany). Sequence quality was assessed, and consensus sequences were generated by assembling forward and reverse reads. Taxonomic identification of isolates was performed through nucleotide BLAST (Basic Local Alignment Search Tool) comparisons against the NCBI GenBank database.

Subsequent analyses were conducted using MEGA v12 [61]. Multiple sequence alignments for each gene region were generated with the CLUSTALW algorithm [62], incorporating both newly obtained sequences and relevant reference sequences retrieved from GenBank (Table S3). For multi-locus phylogenetic analysis, the aligned sequences were concatenated into a single dataset. Phylogenetic relationships were inferred using the Maximum Likelihood (ML) method under the Tamura–Nei model of nucleotide substitution [63]. The robustness of the inferred topology was assessed by bootstrap analysis with 1000 replicates, and the percentage of replicate trees in which the associated taxa clustered together is reported next to the branches [64]. For the heuristic search, the initial tree was selected as the topology with the highest log-likelihood among those generated using the Neighbor-Joining (NJ) method [65] and the Maximum Parsimony (MP) method.

The analysis included 17 nucleotide sequences. A partial deletion approach was applied to exclude positions with less than 95% site coverage, resulting in a final alignment comprising 1067 positions. All evolutionary analyses were conducted in MEGA v12, using up to four parallel computing threads to enhance computational efficiency.

### 4.2. Plant Materials and Experimental Sites and Plot Design

This study was conducted on 18 almond cultivars (Table 3), all grown under uniform agronomic and environmental conditions in the experimental orchard of INRAE, Domaine des Garrigues (9004 Allée des Chênes, Avignon, France). The trees were planted in 2012 at a spacing of 5 m × 2 m, with one replicate tree per cultivar and grafted on GF667. No fungicide treatments were applied throughout the study period, allowing for natural disease development and controlled inoculation experiments.

Three inoculation methods were evaluated in this study: conidial suspension, mycelial plug, and colonized toothpick. All 18 almond cultivars were inoculated using isolates FS, FJS, FB, and LD via the conidial suspension method. The suspension (1 × 10^5^ conidia mL^−1^) was applied as a single drop onto a superficial wound on the shoot surface, which was subsequently sealed with Parafilm to maintain high humidity.

The mycelial plug inoculation method, using isolate FS, was applied to five cultivars: ‘Ferragnès’, ‘Ferrastar’, ‘Ardèchoise’, ‘Tuono’, and ‘Texas’. Agar plugs (0.5 cm in diameter) excised from 7-day-old cultures grown on potato dextrose agar (PDA) were inserted into shallow wounds on the shoots and immediately wrapped with Parafilm to prevent desiccation.

The same five cultivars were also used for the toothpick inoculation method, conducted with isolates FS and FM. sterilized, pre-colonized toothpicks (approximately 2 cm in length) were inserted into 0.5 cm-deep holes made in the shoot tissue. The inoculation sites were sealed with paraffin wax to protect the wound and retain moisture.

For each cultivar and inoculation method, three biological replicates were performed, with individual branches considered as replicates. The experiment was initiated on 3 June 2025, and symptom development was evaluated at 30, 60, and 90 days after inoculation.

Inocula were prepared according to the specific inoculation technique employed. For the conidial suspension technique, fungal isolates (FS, FJS, FB, LD) were grown on PDA plates at 25 ± 1 °C under a 12 h light/dark photoperiod to induce pycnidia development. After 15 days of incubation, mature pycnidia were scraped from the agar surface with a sterile loop and transferred into sterile beakers containing distilled water. The conidia were released using a handheld electric homogenizer, and the resulting suspension was filtered through a 0.45 µm membrane filter (Thermo Fisher Scientific, Waltham, MA, USA) to remove mycelial debris. Serial dilutions were then performed to obtain a final concentration of approximately 1 × 10^5^ conidia mL^−1^.

For the mycelial plug technique, isolates were cultured on PDA plates in complete darkness at 25 ± 1 °C for 7 days to promote vegetative mycelial growth while preventing sporulation. Agar plugs (0.5 cm in diameter) were excised from the actively growing colony margins and used for inoculation.

For the toothpick technique, sterile wooden toothpicks (≈2 cm long) were inserted into PDA plates previously inoculated with the respective fungal isolates and incubated in darkness at 25 °C for 10 days to allow for complete colonization by the mycelium.

### 4.3. Data Analysis

Data were analyzed using JMP^®^ Pro software (version 18; SAS Institute Inc., Cary, NC, USA, 1989–2023). Prior to performing one-way analysis of variance (ANOVA) followed by Tukey–Kramer’s Honestly Significant Difference (HSD) post hoc test, data distributions were assessed for normality (Figure S3). Statistical differences were considered significant at *p* ≤ 0.05 or *p* ≤ 0.01.

Disease symptom data were further analyzed using R software (version 2024.12.1). To determine the optimal number of clusters, the NbClust package was employed, which evaluates multiple clustering criteria simultaneously. Based on this evaluation, k-means clustering was performed with the number of clusters (*k* = 3) as determined by NbClust. The algorithm was executed with the parameter *nstart* = 25, which runs 25 iterations with different centroid initializations to minimize suboptimal convergence. To ensure reproducibility, a fixed random seed was set (*set.seed* (*123*)).

Cluster visualization was performed using the factoextra package and the *fviz_cluster* function, which displays the clusters and their centroids in the plane defined by the first two principal components derived from Principal Component Analysis (PCA).

To evaluate the temporal progression of disease symptoms, boxplots were generated using the ggpubr package in the R environment. Lesion length data (cm), collected at three time points (8 July, 4 August, and 2 September), were converted into long format using the *pivot_longer* function from the tidyr package. The *ggboxplot* function was then applied to visualize lesion length as the dependent variable. A global ANOVA was used to assess statistical differences among the three time points (*stat_compare_means* (*method = “anova”*)), followed by pairwise comparisons using Student’s *t*-test (*stat_compare_means* (*method = “t.test”)*).

## 5. Conclusions

Overall, this study contributes to clarifying the pathogenic variability of *Diaporthe amygdali* and the differential responses of almond cultivars, providing valuable information for genetic improvement programs and for cultivar selection in areas at risk of disease outbreaks. The identification of potentially more tolerant genotypes is essential for mitigating the impact of this emerging disease and for promoting more sustainable management of Mediterranean almond orchards.

Our results further suggest the presence of tolerance-associated genes that could be exploited in breeding programs. Future research should therefore focus on the precise identification of these genes and their introgression into new cultivars using molecular marker–assisted approaches. Given the absence of detectable genetic variability among the analyzed isolates, it can be reasonably assumed that such tolerance traits may confer effectiveness against most *D. amygdali* strains currently present in the surveyed regions.

High-density genotyping using almond SNP arrays [66], combined with phenotypic evaluation of either biparental populations or the core germplasm collection through controlled inoculation with *D. amygdali*, will enable the identification of tolerance loci through molecular mapping or genome-wide association studies (GWAS).

## Figures and Tables

**Table 1:** Origin and host information for *Diaporthe amygdali* isolates recovered from infected almond orchards in France, along with two reference strains (M2 ref 18-286 and M17 ref 18-286) supplied by ANSES LYON (AGENCE NATIONALE DE SÉCURITÉ SANITAIRE de l’alimentation, de l’environnement et du travail). For the orchard location, the French department is indicated in bracket.

Orchard Location	Year of Isolation	Variety	ID
Borgho (2B), Corsica, France	2018	Unknown	M2 ref 18-286
Borgho (2B), Corsica, France	2018	Unknown	M17 ref 18-286
Charolles (26), France	2022	Lauranne	LD
Charolles (26), France	2022	Mandaline	MD
St Didier (84), France	2022	Ferragnès	FS
Manduel (30), France	2022	Ferragnès	FB
Donzere (26), France	2025	Ferragnès	FM
St Didier (84), France	2025	Ferragnès	FJS

**Table 2:** Conidial measurements of the isolates after 20 days of growth on potato dextrose agar (PDA) under a 12 h UV-light photoperiod. Statistically significant differences among isolates are indicated by different letters according to Tukey’s test (*p* < 0.01). Data are reported as mean ± standard error (n = 30).

Conidia				
Width (µm)	Tukey HSD Test	Length (µm)	Tukey HSD Test	Isolate
2.6 ± 0.1	BCD	7.4 ± 0.2	B	FB
3.1 ± 0.1	A	8.7 ± 0.4	A	FJS
2.6 ± 0.1	BCD	6.6 ± 0.3	B	FM
2.7 ± 0.1	ABC	7.2 ± 0.2	B	FS
2.3 ± 0.1	D	7.3 ± 0.3	B	LD
2.5 ± 0.1	BCD	6.9 ± 0.3	B	M2
2.5 ± 0.1	CD	7.9 ± 0.3	AB	M17
2.9 ± 0.1	AB	9.1 ± 0.4	A	MD

**Table 3:** Origin and main characteristics of the 18 almond (*Prunus dulcis*) cultivars included in the study. Cultivars were grown under uniform conditions at INRAE–Domaine des Garrigues (Avignon, France). Susceptibility levels are based on disease response to *Diaporthe amygdali*: S = susceptible, T = tolerant, I = intermediate. “X” indicates cultivars selected for testing the respective inoculation method (conidial suspension, mycelial plug, or toothpick).

Code No.	Variety	Country *	Flowering Season **	Resistance Level to *D. amygdali*	Inoculum Method ***		
					A	B	C
R486	Ferragnès	FRA	4	S	X	X	X
R1877	Hybride R1877	FRA	5		X		
R916	Lauranne	FRA	5		X		
R800	Ferrastar	FRA	5	T	X	X	X
R61	Ardèchoise	FRA	3	T	X	X	X
R185	Marcona	ESP	2		X		
R1046	Princesse-JR	FRA	2		X		
R934	Guara	ITA	5		X		
R998	Mandaline	FRA	5		X		
R219	Tuono	ITA	4	S	X	X	X
R993	Hybride R993	FRA	5		X		
R1590	Le plan1	FRA	4		X		
R1568	Hybride R1568	FRA	5		X		
R270	Texas	USA	4	T	X	X	X
R1413	Hybride R1413	FRA	7		X		
R1542	Hybride R1542	FRA	5		X		
R860	Filippo Ceo	ITA	4	I	X		
R1591	Le plan2	FRA	5		X		

* Country: FRA = France; ITA = Italy; ESP = Spain; USA = United State of America. ** Flowering season: 1 = early; 2 = medium-early; 3 = intermediate; 4 = medium-late; 5 = late. *** Inoculation made using: A, Conidial suspension; B, Mycelial plug; C, Toothpick.

## Data Availability

The partial sequences of the five amplified genes are currently being registered in the NCBI database.

## Supplementary Materials

The following supporting information can be downloaded at https://www.mdpi.com/article/10.3390/plants15010165/s1: Figure S1. Daily growth rate (cm/day) of *Diaporthe amygdali* colonies grown on PDA at 25 ± 1 °C. Data represent mean ± standard error (n = 3); Table S1: Mean lesion length (cm) of the three clusters of almond varieties across three consecutive disease evaluation surveys (7 July, 4 August, and 2 September). Cluster 1 represents tolerant varieties, characterized by low lesion values and limited progression over time; Cluster 2 includes intermediate varieties showing moderate lesions and relatively stable conditions; and Cluster 3 comprises susceptible varieties with extensive lesions and a rapid increase over time. The three clusters were identified by k-means analysis (*k* = 3). Data are reported as mean ± standard error; Table S2: Primer sets used in the work; Table S3: GenBank references sequences used for phylogenetic analyses; Figure S2. Phylogenetic tree showing the relationships among *Diaporthe* species and *D. amygdali* isolates collected from different regions of southern France. The tree was constructed using the Maximum Likelihood (ML) method under the Tamura–Nei substitution model, with a scale bar representing 0.1 nucleotide substitutions per site. Phylogenetic tree based on sequences of *cal* gene (A); *his* gene (B); partial ITS region (C); *tub2* gene (D); *tef 1-α* gene (E); Figure S3. Distribution of the data used in the ANOVA. (A–C) Data used for the ANOVA of measurements taken on branches inoculated with the conidial suspension, including all varieties and isolates, corresponding to the first, second, and third assessments, respectively. (D) Data used in the ANOVA for differences in lesion diameter among isolates. (E) Data used in the ANOVA assessing the differential behavior of the isolates in branch inoculations with the conidial suspension, including all varieties. (F–H) Data used in the ANOVA comparing the different inoculation methods, considering only the ‘Ardèchoise’, ‘Ferragnès’, ‘Ferrastar’, ‘Texas’, and ‘Tuono’ cultivars and the FS isolate. (I,J) Data used in the ANOVA for the length and width of α-conidia for each isolate. The table reports the W value and *p*-value obtained from the Shapiro–Wilk test for each dataset.
