# Supplementary material for: Study on the Susceptibility of Some Almond (Prunus dulcis) Cultivars to the Pathogen Diaporthe amygdali
Source: Plants (Basel). 2026 Jan 5;15(1):165. doi: 10.3390/plants15010165 (PMC12787381; doi:10.3390/plants15010165)
Supplement: Supplementary file 1 [file plants-15-00165-s001.zip › plants-3974852-supplementary.pdf]

Supplementary Materials: The following supporting information can be downloaded at: https://www.mdpi.com/article/doi/s1.

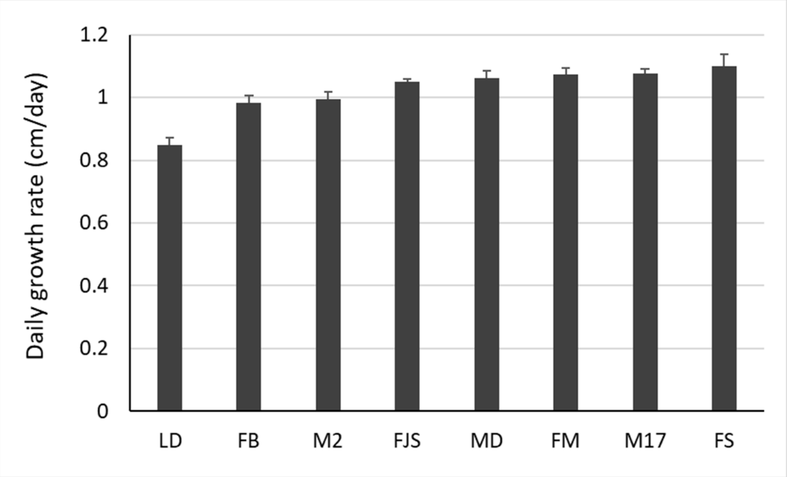

Figure S1. Daily growth rate (cm/day) of *Diaporthe amygdali* colonies grown on PDA at 25± 1 °C. Data represent mean ± standard error (n = 3)

Table S1: Mean lesion length (cm) of the three clusters of almond varieties across three consecutive disease evaluation surveys (7 July, 4 August, and 2 September). Cluster 1 represents tolerant varieties, characterized by low lesion values and limited progression over time; Cluster 2 includes intermediate varieties showing moderate lesions and relatively stable conditions; and Cluster 3 comprises susceptible varieties with extensive lesions and a rapid increase over time. The three clusters were identified by k-means analysis (*k*=3). Data are reported as mean±standard error.

Cluster	1st survey 7_08_2025 (cm)	2nd survey 8_04_2025 (cm)	3rd survey 9_02_2025 (cm)	Description
1	0.332±0.049	0.464±0.033	0.511±0.027	Tolerant — low values and limited growth over time
2	0.510±0.059	0.605±0.060	0.785±0.037	Intermediate — moderate lesions, stable condition
3	0.758±0.062	0.900±0.056	1.380±0.068	Susceptible — extensive lesions and rapidly increasing over time

Table S2: Primer sets used in the work;

Target region	Name	Sequence 5'-3'	Annealing Temperature (°C)	Reference
ITS	ITS1_F	TCCGTAGGTGAACCTGCGC	55	[54]
ITS	ITS4_R	TCCTCCGCTTATTGATATGC		
tef1-α	EF1-688F	CGGTCACTTGATCTACAAGTGC	55	[55]
tef1-α	EF1-1251R	CCTCGAACTCACCAGTACCG		
β-tub	BtCadF	MATGCGTGAAATYGTAAGT	50	[56]
β-tub	BtCadR	TCAGCACCCTCAGTGTAATG		
his3	CYLH3_F	AGGTCCACTGGTGGCAAG	59	[57]
his3	H3-1b	GCGGGCGAGCTGGATGTCCTT		
CAL	CL1C	GAATTCAAGGAGGCCTTCTC	58	[58]
CAL	CL2C	CTTCTGCATCATGAGCTGGAC		

Table S3: GenBank references sequences used for phylogenetic analyses.

**Species**	**Strain number**	**Country and host**	**GenBank/EBI accession number**				
			**ITS**	**CAL**	**TUB2**	**HIS3**	**TEF-1 α**
Diaporthe amygdali	CBS126680	Portugal	MH864209	KC343265	KC343991	KC343507	KC343749
Diaporthe mediterranea	DAL-34	Alicante, *Prunus dulcis*	MT007489	MT006761	MT006686	MT007095	MT006989
Diaporthe sterilis	CBS136969	Italy, *Vaccinium corymbosum*	NR_152459	KJ160548	KJ160528	MF418350	KJ160611
Diaporthe kadsurae	CFCC52587	China, *Kadsura longipedunculata*	MH121522	MH121440	MH121601	MH121480	MH121564
Diaporthe chongqingensis	PSCG435	China, *Pyrus pyrifolia*	MK626916	MK691209	MK691321	/	/
Diaporthe acaciigena	CBS129521	Australia, *Acacia retinodes*	NR_137113	KC343247	KC343973	KC343489	KC343731
Diaporthe rudis	CBS113201	Portugal, *Sphaeria rudis*	MH862916	KC343476	KC344202	KC343718	KC343960
Diaporthe ambigua	CBS114015	South Africa	MH862953	KC343252	KC343978	KC343494	KC343736
Diaporthe malorum	CAA734	Portugal, *Malus domestica*	KY435638	KY435658	KY435668	KY435648	KY435627
Diaporthella corylina	CBS 121124	China, *Corylus* sp.	KC343004	KC343246	KC343972	KC343488	KC343730

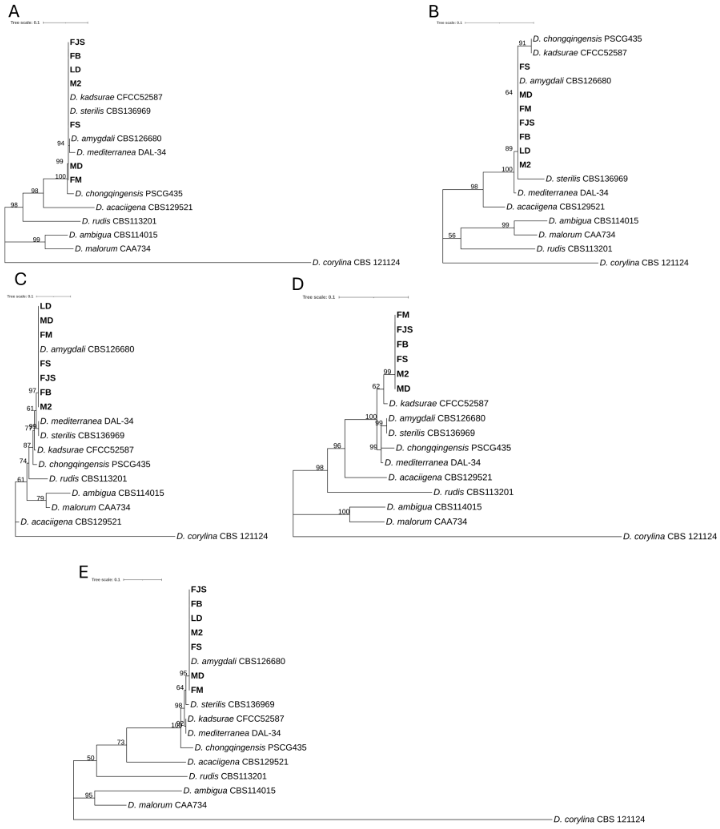

Figure S2. Phylogenetic tree showing the relationships among *Diaporthe* species and *D. amygdali* isolates collected from different regions of southern France. The tree was constructed using the Maximum Likelihood (ML) method under the Tamura–Nei substitution model, with a scale bar representing 0.1 nucleotide substitutions per site. Phylogenetic tree based on sequences of *cal* gene (A); *his* gene (B); partial ITS region (C); *tub2* gene (D); *tef 1-α* gene (E).

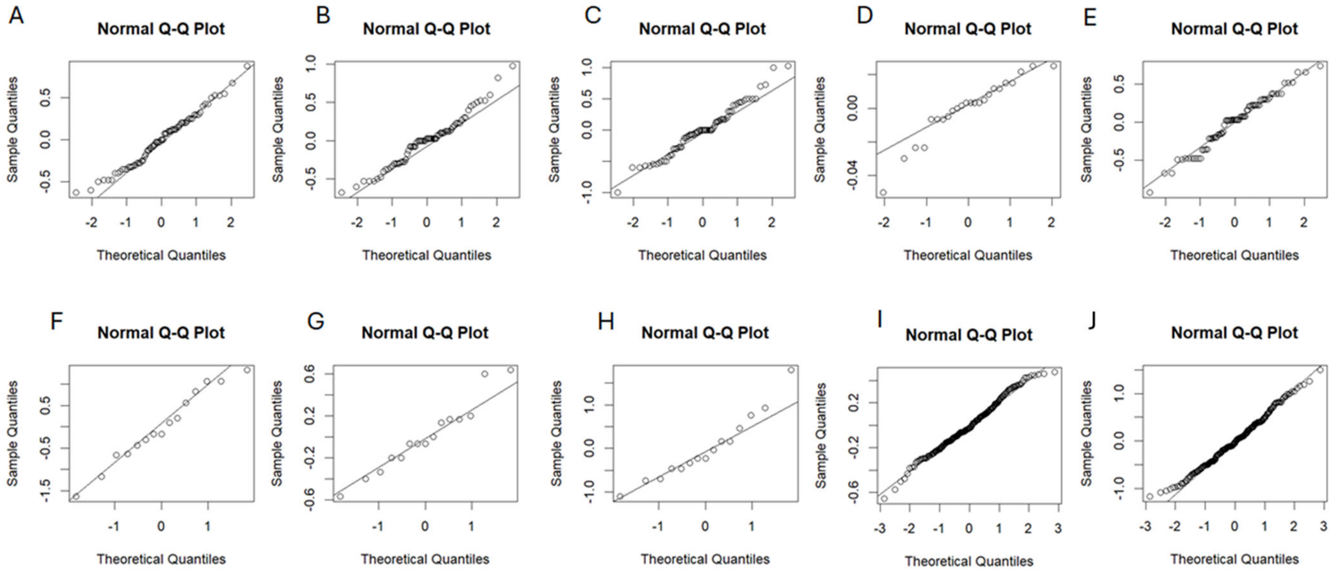

	Shapiro-Wilk normality test									
	(A) 1st survey 7_08	(B) 2nd survey 8_04	(C) 3rd survey 9_02	(D) Diameter isolate	(E) Strain	(F) Conidic suspension	(G) Mycelium plug	(H) Toothpick	(I) α- conidia length	(J) α- conidia width
W	0.98	0.97	0.98	0.92	0.98	0.97	0.96	0.94	0.99	0.99
p-value	0.48	0.09	0.21	0.054	0.21	0.86	0.61	0.42	0.13	0.28

Figure S3. Distribution of data used in ANOVA. A, B, C) Data used for ANOVA of measurements taken on branches inoculated with the conidial suspension, including all varieties and isolates used, first, second and third measurements respectively; D) Data used in ANOVA for the difference in diameter of the respective isolates; E) Data used in ANOVA for the different behaviour of the isolates in the inoculation on branches with the conidial suspension, including all varieties; F,G,H) Data used in the ANOVA for the different inoculation methods used, considering only the Ardèchoise, Ferragnès, Ferrastar, Texas and Tuono varieties, and the FS isolate; I,J) Data used in the ANOVA for length and width of α-conidia for each isolate.

In the table, the W value and p-value obtained with the Shapiro-Wilk test are reported for each of the data.
